# Moderate intermittent hypoxic conditioning to enhance vascular function and cardiorespiratory fitness in the elderly: A randomized controlled trial

**DOI:** 10.14814/phy2.70432

**Published:** 2025-10-09

**Authors:** Hugo Randy, Titouan P. Perrin, Abdallah Ghaith, Dario Kohlbrenner, Patrice Flore, Benoit Champigneulle, Michel Guinot, Stéphane Doutreleau, Julien Brugniaux, Samuel Verges, Mathieu Marillier

**Affiliations:** ^1^ Université Grenoble Alpes, Inserm, CHU Grenoble Alpes, HP2 Grenoble France

**Keywords:** cardiorespiratory fitness, endothelial function, flow‐mediated dilation, intermittent hypoxic conditioning, vascular aging

## Abstract

Vascular aging involves reduced endothelial function, a key factor in cardiovascular diseases. Intermittent hypoxia may improve endothelial function and cardiorespiratory fitness (CRF), but its effects in elderly individuals, especially in the mid‐term, have not yet been studied. This randomized, single‐blind controlled trial aimed to investigate whether an 8‐week intermittent hypoxic conditioning (IHC) program may enhance flow‐mediated dilation (FMD) and CRF in elderly individuals. Twenty‐six participants (60–80 year‐old) were assigned to either the IHC (*n* = 12) or the control group (CTL: *n* = 14). The IHC group underwent 24 passive intermittent hypoxia sessions (3/week). Brachial artery FMD, cardiopulmonary exercise testing (CPET), and ambulatory 24‐h blood pressure were assessed at baseline (Pre), immediately post‐intervention (Post 1), and 2 months later (Post 2). FMD showed a trend toward improvement in the IHC group, being significant when normalized for baseline artery diameter (*p* = 0.023; *η*_*p*_^2^ = 0.150) between Pre and Post 2. Peak ventilation during CPET increased from Pre to Post 1 (*p* = 0.021), with no other significant CRF changes. Daytime systolic blood pressure decreased by 6 mmHg (*p* = 0.070, *η*_*p*_^2^ = 0.105). No significant alterations in these outcomes were observed in the CTL group (*p* > 0.05). Moderate IHC enhanced mid‐term endothelial function, suggesting potential to mitigate age‐related vascular decline.

## 1 INTRODUCTION

Cardiovascular health is a major public health concern, particularly affecting elderly individuals (Climie et al., 2023; Timmis et al., 2022). The aging vascular system is characterized by structural and functional changes that contribute significantly to the overall cardiovascular burden (Ungvari et al., 2018). Endothelial dysfunction, a hallmark of vascular aging, typically associated with a downregulation in the production of vasomotor substances (e.g., nitric oxide, NO) by endothelial cells (ECs) (Daiber et al., 2017) is often associated with the progression of clinical conditions such as atherosclerosis and arteriosclerosis, both of which significantly increase the risk of stroke and myocardial infarction (Gutiérrez et al., 2013; Numazaki et al., 2023).

Brachial flow‐mediated dilation (FMD) is currently the most widely used non‐invasive technique to assess endothelial function (Heiss et al., 2023) and is primarily mediated by the release of nitric oxide (NO) from endothelial cells (Green et al., 2014). Flow‐mediated dilation is known to decline with aging (Celermajer et al., 1994), various clinical conditions (Mućka et al., 2022) and sedentary lifestyles (Black et al., 2009). This impairment has also been observed in individuals with traditional cardiovascular risk factors, such as smoking (Johnson et al., 2010), arterial hypertension (Moriguchi et al., 2005) or low cardiorespiratory fitness (CRF) (Buscemi et al., 2013). Moreover, FMD has been strongly associated with the risk of future cardiovascular events, making it a valuable marker of cardiovascular health (Green et al., 2011; Yeboah et al., 2007).

Given the pivotal role played by vascular function in the development of cardiovascular disease, clinical research has focused on strategies to prevent or counteract this age‐related deterioration. In recent decades, repeated exposure to intermittent hypoxia—also known as intermittent hypoxic conditioning (IHC)—has emerged as a promising therapeutic approach with potential cardioprotective effects (Mallet et al., 2018). For instance, Panza et al. (2022) demonstrated that, in untreated hypertensive men with obstructive sleep apnea, continuous positive airway pressure (CPAP) treatment combined with daily exposure to mild IHC (5 days per week over 3 weeks, comprising twelves 2‐min hypoxic cycles at 8% FiO_2_, interspersed with 2‐min normoxic cycles) significantly reduced systolic blood pressure, compared to control treatment with CPAP alone. Similarly, Lyamina et al. (2011) reported that a 20‐day consecutive protocol of IHC (four to ten 3‐min hypoxic cycles at 10% FiO_2_ interspersed with 3‐min normoxic cycles) resulted in an inverse correlation between reductions in systolic blood pressure and increases in urinary NO metabolites, with patients normalizing arterial blood pressure (ABP) and maintaining normotension for at least 3 months post‐intervention. Additionally, IHC, with or without exercise, has been shown to improve exercise tolerance in various populations (Lizamore & Hamlin, 2017). Notably, Shatilo et al. (2008) demonstrated that in sedentary elderly individuals, a 10‐day IHC protocol involving daily sessions (comprising four 5‐min hypoxic cycles at 12% FiO_2_, interspersed with 5‐min normoxic cycles) led to significant improvements in various exercise tolerance markers, including enhanced submaximal workload capacity, anaerobic threshold, and lower heart rate at a given submaximal workload.

It is well‐established that individuals exposed to chronic intermittent or continuous severe hypoxia (such as those with sleep apnea or high‐altitude residents) exhibit markers of endothelial dysfunction (Savina et al., 2024; Wang et al., 2015). In contrast, only a few studies have investigated the potential benefits of acute moderate intermittent hypoxia (IH) on endothelial function. For instance, Hanson et al. (Hanson et al., 2022) reported that IH (five 6‐min hypoxic cycles targeting 80% SpO_2_, interspersed with 4‐min normoxic cycles) can mitigate the decline in FMD typically observed after short periods of inactivity. Conversely, Stray‐Gundersen et al. (2024) reported no significant effects of IH (eight 4‐min hypoxic cycles targeting 80% SpO_2_, interspersed with resaturation bouts) on FMD in either young adults or elderly individuals. Despite these mixed results, it is important to emphasize that the above‐mentioned studies were conducted in acute settings (i.e., following a single IH session), with distinct hypoxic administration patterns. The potential value of repeating moderate IH to improve endothelial function remains to be investigated.

Considering that elderly individuals are particularly prone to developing cardiovascular diseases, it seems reasonable that they would benefit from IHC. Additionally, since cardiorespiratory fitness (CRF) is a key marker of healthy aging (Blaha et al., 2016) and is associated with greater FMD (Buscemi et al., 2013), it seems pertinent to investigate whether IHC may also improve this outcome. We, therefore, aimed to examine the effects of an 8‐week IHC program on brachial artery FMD and CRF in elderly individuals. We hypothesized that IHC would improve both peripheral endothelial function and CRF markers. Lastly, we expected that these positive effects would persist for at least 2 months based on the above‐mentioned literature.

## 2 MATERIALS AND METHODS

### 2.1 Participants

**FIGURE 1:**
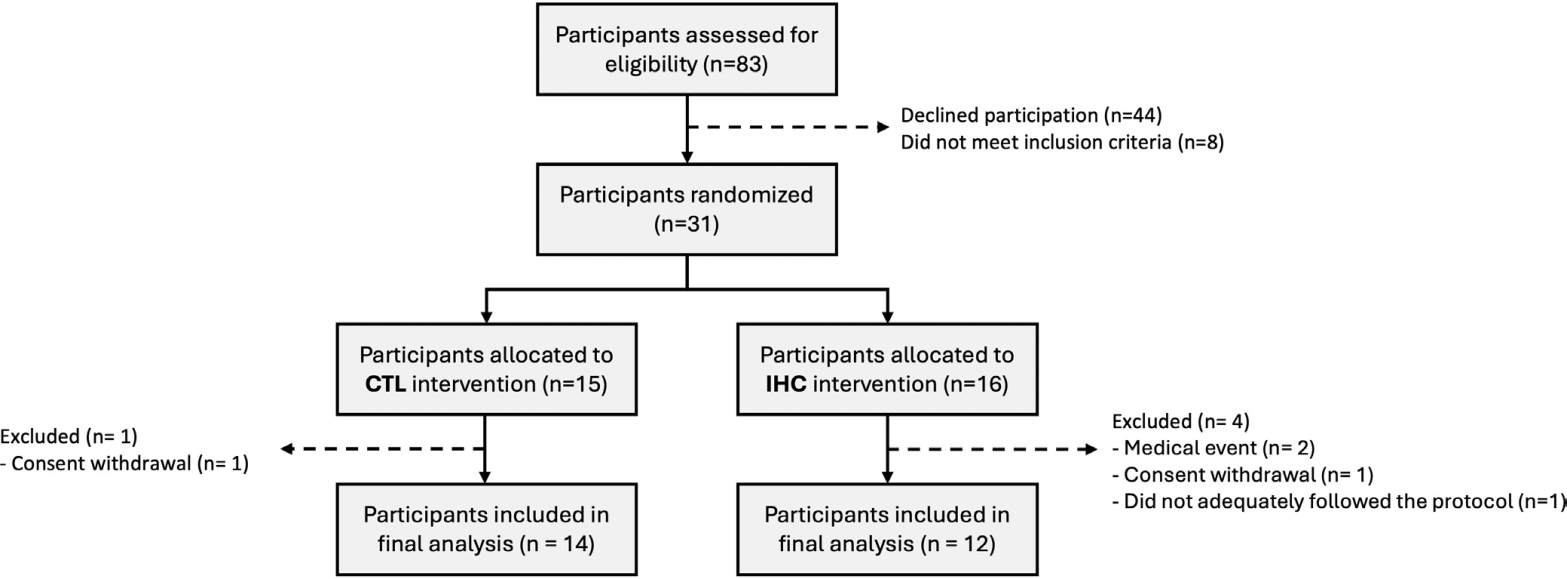
Flow chart illustrating participants' enrollment. CTL, control group; IHC, intermittent hypoxic conditioning group.

Thirty‐one Caucasian elderly individuals were included in this monocentric, single‐blind, parallel group, randomized controlled trial, among which 26 completed the study (see Figure 1 for further details on participants' enrolment). Participants were recruited from different elderly associations in the geographical area of Grenoble (France) or by word of mouth from October 2021 to May 2024. No socioeconomic considerations drove the recruitment. Participants were deemed eligible for study inclusion as per the following criteria: (i) aged 60–80 years; (ii) body‐mass index <30 kg/m^2^; and (iii) no chronic cardiovascular, metabolic, or respiratory diseases (except conditions commonly associated with aging, that is, arterial hypertension and treated sleep apnea). In female participants, menopausal status was not formally assessed. However, given that the youngest woman was 60 years old, it is highly likely that all were indeed postmenopausal. Most participants were recreationally active, but none were engaged in structured exercise training. Prior to their inclusion, participants underwent a clinical examination, including a 12‐lead electrocardiogram and spirometry.

### 2.2 Experimental design

**FIGURE 2:**
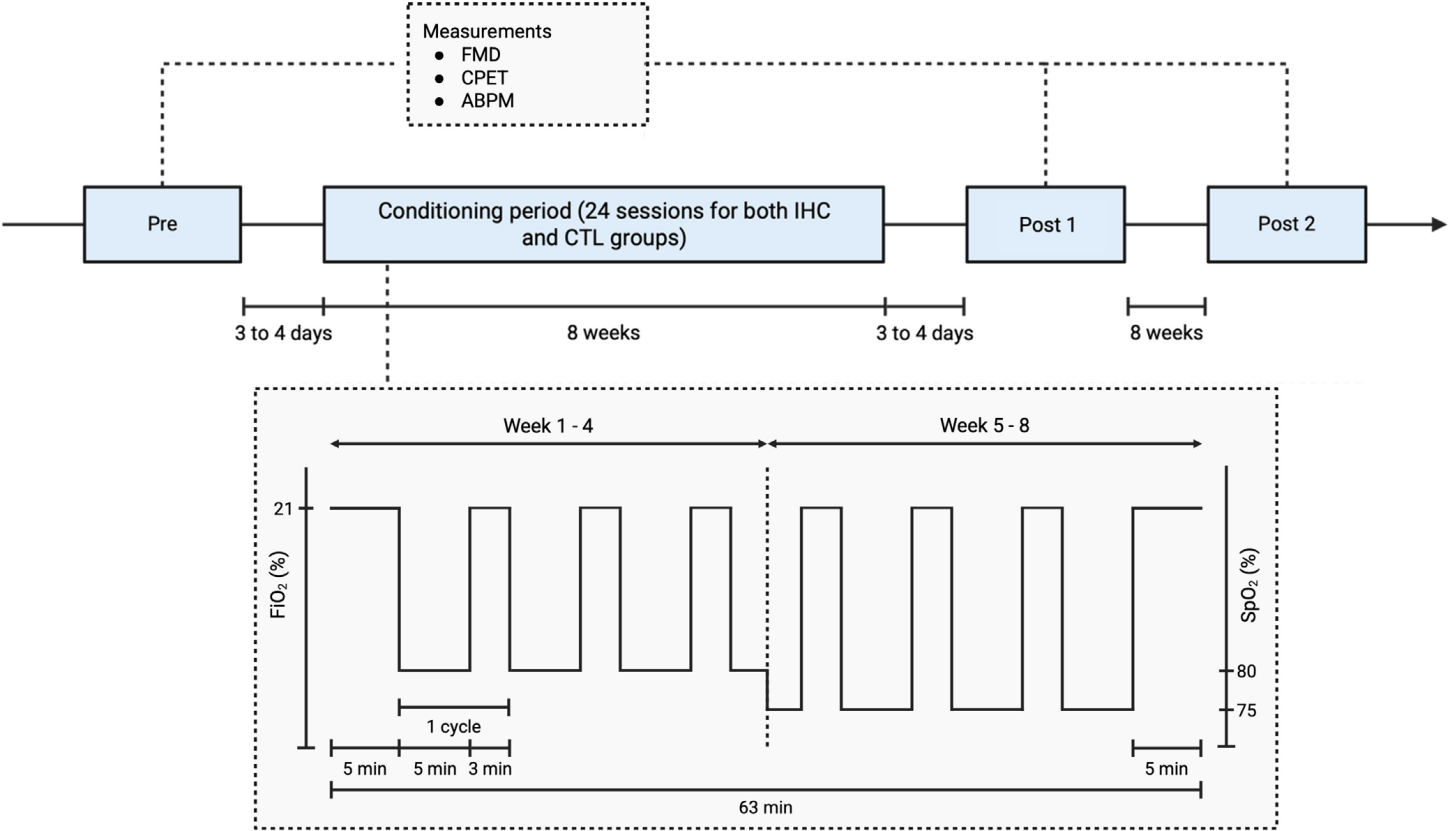
Outline of study protocol. The first (Pre) and second (Post 1) experimental visits were performed 3–4 days before and after the conditioning period, respectively. The final experimental visit (Post 2) was conducted 8 weeks after the second visit. Participants were either allocated to intermittent hypoxic conditioning (IHC) or control group (CTL). The conditioning period included 24 sessions evenly distributed over 8 weeks (3 sessions/week). Each IHC session included seven cycles, each cycle consisting of 5 min of hypoxia followed by 3 min of normoxia. The initial hypoxic stimulus was individually adjusted to achieve a target oxygen saturation by pulse oximetry (SpO_2_) of 80%. Starting from week 5, the severity of the stimulus was increased to reach an SpO_2_ of 75%. During CTL sessions, participants breathed sham hypoxia for the same amount of time as those in the IHC group. ABPM, ambulatory blood pressure monitoring; CPET, cardiopulmonary exercise test; FiO_2_, inspired fraction in O_2_; FMD, flow mediated dilation.

Following inclusion and the first experimental session, participants were randomized to either the IHC group (experimental condition) or the sham normoxic group (control condition, CTL). The process was performed with a randomization list (1:1 allocation ratio) by an investigator not involved in experimental measures. Participants were blinded to the allocated condition. Each participant completed three conditioning (or sham) sessions per week over an 8‐week period. Experimental measurements—including macrovascular, cardiopulmonary exercise test (CPET) and ambulatory blood pressure monitoring (ABPM)—were conducted at three time points: prior to the conditioning period (Pre), 3–4 days following the end of the intervention (Post 1), and 2 months post‐intervention (Post 2) (Figure 2). To minimize the impact of diurnal variation, experimental sessions were conducted at the same time of day (i.e., starting at 8–9 am or 1–2 pm) for each participant.

### 2.3 Conditioning sessions

During sessions, participants were comfortably seated in a semi‐recumbent position in a quiet environment. Heart rate and SpO_2_ were continuously recorded for every session and participant. To maintain blinding during all conditioning sessions, a windscreen was used to hide the gas delivery setup, and participants were blind to SpO_2_ readings.

#### 2.3.1 Intermittent hypoxic conditioning sessions

Sessions began with a 5‐min baseline period while breathing room air, followed by seven cycles of hypoxia, each lasting 5 min interspersed with 3‐min periods of normoxic breathing (Figure 2). Using a gas‐mixing device (Altitrainer®, SMTEC S.A., Nyon, Switzerland), the inspired fraction of oxygen (O_2_) was individually adjusted during each session to achieve a target arterial O_2_ saturation assessed by pulse oximetry (SpO_2_ = 80% from week 1 to week 4; 75% from week 5 to week 8) (Radical‐7®, Massimo corporation, Irvine, CA, USA). Each session ended with a 5‐min normoxic phase, allowing participants to return to their pre‐session physiological state. In total, each session lasted 63 min, including 35 min of hypoxic breathing. This time pattern and severity of hypoxic stimulus are based on a previous study from our laboratory that demonstrated beneficial effects (Chacaroun, Borowik, Doutreleau, et al., 2020).

#### 2.3.2 Normoxic sessions

During normoxic sessions, participants breathed room air (inspired fraction of O_2_ = 0.21) delivered through a sham gas‐mixing device (Altitrainer placebo®, SMTEC S.A., Nyon, Switzerland) for the same duration as those in the IHC group.

### 2.4 Experimental measurements

During each experimental visit (Pre, Post 1 and Post 2), vascular function was assessed using FMD and nitrate‐mediated dilation (NMD) measurements. Following these assessments, participants underwent a CPET and were subsequently fitted with an ABPM before leaving the laboratory.

#### 2.4.1 Macrovascular function

##### Flow‐mediated dilation

Flow‐mediated dilation assessment was conducted following the current guidelines (except fasting due to the CPET performed afterward) (Thijssen et al., 2019) by the same experienced operator. After 20 min of supine rest in a quiet, temperature‐controlled room, FMD of the right brachial artery was measured using a high‐resolution duplex ultrasound system (Terason 3200t, Teratech Corporation, Burlington, VE, USA) with a 15–4 MHz linear array transducer secured with a stereotactic adjustable clamp (MP‐PH0001®, Hitachi‐Aloka Medical Ltd., Tokyo, Japan). Following a 1‐min baseline measurement, an occlusion cuff (placed around the forearm, proximal to the olecranon joint) was inflated to a suprasystolic pressure (240 mmHg) for 5 min and then quickly released. Brachial artery diameter and blood flow velocity were continuously recorded from baseline until 5 min post‐deflation.

##### Nitrate‐mediated dilation

After an additional 20 min of supine rest, NMD was assessed. Brachial artery diameter and blood flow velocity were again recorded during a 1‐min baseline period. A single sublingual dose of glyceryl trinitrate (Natispray, 300 μg, Teofarma, Pavia, Italy) was then administered, followed by an 8‐min continuous recording of the brachial artery diameter and blood flow velocity.

All FMD and NMD measurements were recorded at 30 Hz with a dedicated software (Camtasia, TechSmith Corporation, East Lansing, MI, USA) and saved for offline analysis with edge detection and wall tracking software (Bloodflow Analysis; National instruments, Austin, TX, USA). Based on the image quality (i.e., clear distinction between the artery wall and the lumen), a region of interest was identified. Outcomes for both tests were defined as the maximal diameter change (expressed in percentage) from baseline (*D*_BASE_) to peak (*D*_PEAK_) dilation during the post‐deflation reactive hyperemia during the FMD test or after sublingual glyceryl trinitrate administration during the NMD test. Shear rate was automatically calculated as [(4 × velocity)/diameter] and used for determining shear rate area under the curve (SR_AUC_). To account for inter‐individual baseline diameter differences, FMD was allometrically scaled (see Section 2.7 for further details on the procedure).

#### 2.4.2 Cardiopulmonary exercise test

Participants performed an incremental CPET to voluntary exhaustion on an electronically braked cycle ergometer (Ergoselect 100P, Ergoline GmbH, Bitz, Germany). Each test began with a 3‐min rest period for steady‐state baseline measurements, after which the initial power output was set at 30 W for men and 20 W for women. The workload was then increased by 10 W every minute (for both men and women) until exhaustion. Metabolic and ventilatory parameters were continuously recorded using a breath‐by‐breath ergospirometer (Metamax 3B, Cortex Biophysik GmbH, Leipzig, Germany). Heart rate was monitored with a 12‐lead electrocardiogram (Custo cardio 110 BT; Custo med GmbH, Ottobrunn, Germany), while SpO_2_ was measured with a pulse oximeter at the fingertip (Nonin® 3150 WristOx2, Plymouth, MN, USA). Dyspnea and leg discomfort scores were recorded every 2 min using the 0–10 category‐ratio Borg scale. Data were extracted as 30‐sec averages from breath‐by‐breath measurements for subsequent data interpretation (Marillier et al., 2023); peak exercise was defined as the last 30 s before exercise cessation. Maximal heart rate, peak absolute O_2_ uptake ($\dot{V}O_{2\text{peak}}$) and work rate were expressed as percent predicted values using the reference equations from Wasserman et al. (Wasserman et al., 2011).

#### 2.4.3 24‐hour ambulatory blood pressure monitoring

Participants were equipped with a blood pressure cuff (Diasys 3+, Novacor, Rueil‐Malmaison, France) on the non‐dominant arm. Arterial blood pressure was monitored every 15 min during daytime (7 am–10 pm) and every 30 min at nighttime (10 pm–7 am) to minimize sleep disturbance. Systolic (SBP) and diastolic (DBP) blood pressure were averaged for daytime, nighttime, and 24‐h periods. Mean arterial pressure (MAP) was calculated for the same periods as [(SBP + (2 × DBP))/3].

### 2.5 Statistical analysis

This research is part of a larger project aiming at investigating the physiological effects of IHC on (cerebro‐)vascular function in elderly individuals. In this context, our sample size calculation was based on an expected 5 ± 4% difference in CO_2_ reactivity of the middle cerebral artery velocity between the hypoxic and normoxic conditions. With *α* = 0.05 and a two‐tailed test of significance, 14 participants per group were required to provide a statistical power of 80% (with nQuery Advisor 7.0, Statistical Solutions, Saugus, MA, USA). All statistical analyses were performed using R software (version 4.1.2, The R Foundation for Statistical Computing, Vienna, Austria). Graphical representations were created with Prism (v. 10.2.2; GraphPad Software, La Jolla, CA, USA). Data distribution was assessed using the Shapiro–Wilk normality test. Participant characteristics were compared using independent *t*‐tests or Mann–Whitney *U*‐tests where appropriate; a Fisher's exact test was applied to compare sex frequencies. For repeated measurements between groups, homogeneity of variance was assessed with Levene's test. For normally distributed data, a two‐way repeated‐measures ANOVA was performed with group (CTL and IHC) and time (Pre, Post 1 and Post 2) as factors with repetition on the last factor (time). Bonferroni post hoc tests were applied to adjust for multiple comparisons when a significant main or interaction effect was achieved. For non‐normally distributed data, within‐group comparisons were made using a Friedman's ANOVA, with a Durbin‐Conover test for pairwise comparisons. A Mann–Whitney *U*‐test was used for between‐group comparisons. Effect sizes for two‐way repeated‐measures and mixed ANOVA were reported using partial eta‐squared (𝜂_𝑝_^2^). Both were interpreted as small effect: 0.01 < 𝜂_𝑝_^2^ < 0.06; moderate effect: 0.06 < 𝜂_𝑝_^2^ < 0.14; large effect: 𝜂_𝑝_^2^ > 0.14 (Cohen, 1988). For Friedman's ANOVA, effect sizes were calculated using Kendall's *W* and interpreted following Cohen's *d* guidelines (small effect: 0.2 < *d* < 0.5; moderate effect: 0.5 < *d* < 0.8; large effect: *d* > 0.8) (Cohen, 1988). Despite different data distribution and analysis, data are presented as mean ± standard deviation (SD) for clarity purpose. All tests were two‐sided and statistical significance was set at *p* < 0.05. Analyses were conducted using a per‐protocol approach.

### 2.6 Allometric scaling of FMD

According to the method described by Atkinson et al. (2013), allometrically scaled FMD was analyzed via a mixed two‐way ANOVA with the diameter change on the natural log scale (ln*D*_PEAK_ − ln*D*_BASE_) as the dependent variable, group and visit as fixed factors, and log‐transformed baseline diameter (ln*D*_BASE_) as a covariate. Tukey post hoc tests were used for pairwise comparisons. To calculate individual scaled FMD values, we first performed a linear regression to determine the slope (*β* coefficient) of the relationship between the natural log of peak diameter (ln*D*_PEAK_, dependent variable) and baseline diameter (ln*D*_BASE_, independent variable), separately for each group and across all time points. Scaled FMD was then computed for each participant using the following formula:

$$\left(\frac{\ln D_{\text{PEAK}}}{\ln{D_{\text{BASE}}}^{\beta }}-1\right)\times 100.$$

## 3 RESULTS

### 3.1 Participant characteristics

Thirty‐one participants were included in the study and randomized to the CTL or IHC intervention; 26 completed the study and were included in the final analysis. Among the 5 excluded participants, two discontinued due to medical reasons unrelated to the protocol (acute pneumonia and initially unreported vascular disease), two withdrew their consent, and one did not adequately follow the IHC protocol. There were no significant differences between the two groups in terms of sex distribution, anthropometric data, or pulmonary function parameters (Table 1; all *p* > 0.05). Two participants had hypertension (controlled with perindopril or lercanidipine), three were treated with continuous positive airway pressure for obstructive sleep apnea, and two had glaucoma. All the participants were already being treated for their condition (see Table 1 for further details). No adverse events related to the IHC protocol were observed throughout the study.

**TABLE 1:** Characteristics of the participants.

	CTL (*N* = 14)	IHC (*N* = 12)
Anthropometry		
Women/men	9/5	5/7
Age (years)	70 ± 6	70 ± 6
Height (cm)	165 ± 10	167 ± 8
Body mass (kg)	68 ± 12	66 ± 13
Body‐mass index (kg/m^2^)	24.9 ± 2.9	23.3 ± 3.5
Pulmonary function		
FEV_1_ (% pred)	121 ± 16	115 ± 16
FVC (% pred)	119 ± 16	112 ± 16
FEV_1_/FVC ratio (% pred)	108 ± 6	105 ± 7
Treatments		
CPAP	2	1
ACE inhibitor	1	—
Calcium antagonist	—	1
Antiglaucoma agent	1	1
Diuretic	1	—

*Note*: Data are mean ± SD.

Abbreviations: ACE inhibitor, angiotensin‐converting enzyme inhibitor; CPAP, continuous positive airway pressure; CTL, control group; FEV_1_, forced expiratory volume in 1 s; FVC, forced vital capacity; IHC, intermittent hypoxic conditioning group.

### 3.2 Flow‐mediated dilation

**FIGURE 3:**
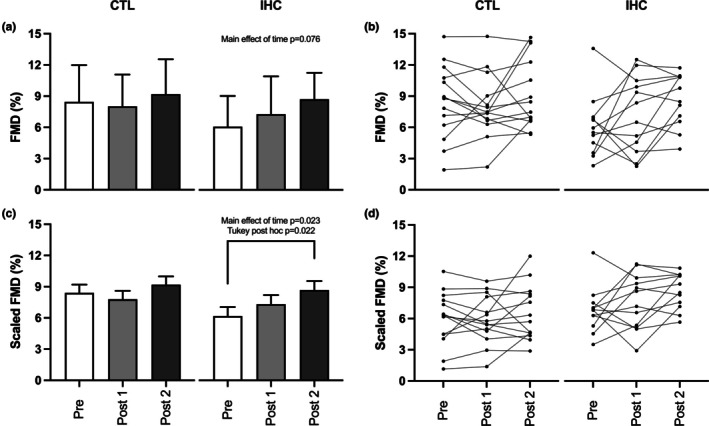
Flow‐mediated dilation (FMD) and allometrically scaled FMD measured at three time points (Pre, Post 1, and Post 2) in the Control (CTL) and Intermittent Hypoxic Conditioning (IHC) groups. Panels (a) and (b) show group means and individual data points for FMD, respectively. Panels (c) and (d) displays group means and individual data points for scaled FMD, respectively. Group data are expressed as mean ± SD. For FMD (panel a), intra‐group comparisons were analyzed using Friedman's ANOVA with multiple pairwise comparisons (Durbin‐Conover). Between‐group comparisons were conducted using Mann–Whitney *U*‐tests. Scaled FMD (panel c) was analyzed with a mixed two‐way ANOVA on log‐transformed diameter changes (ln*D*_PEAK_ − ln*D*_BASE_) as the dependent variable, with group and visit as fixed factors and log‐transformed baseline diameter (ln*D*_BASE_) as a covariate. Tukey post hoc tests were applied for pairwise comparisons, as we observed significant main effect of time (*p* = 0.023; 𝜂_𝑝_^2^ = 0.150).

Flow‐mediated dilation data are depicted in Table 2 and Figure 3. In the IHC group, both *D*_BASE_ and *D*_PEAK_ remained stable throughout the study (*p* > 0.05; Table 2). In contrast, a significant effect of time (*D*_BASE_: *p* = 0.005, *W* = 0.379; *D*_PEAK_: *p* = 0.005, *W* = 0.379) was observed in the CTL group; these measurements were significantly lower at Post 1 compared to Pre (*D*_BASE_: *p* = 0.01; *D*_PEAK_: *p* = 0.01) and Post 2 (*D*_BASE_: *p* < 0.001; *D*_PEAK_: *p* < 0.001). Despite this, the absolute change in artery diameter (*D*_DIFF_ = *D*_PEAK_ − *D*_BASE_) remained constant and similar in both groups (*p* > 0.05). When expressed in relative artery diameter changes, FMD (%) showed a close, but non‐significant (main effect of time: *p* = 0.076; *W* = 0.216) increase in the IHC group from Pre (6.1 ± 3.0%) to Post 2 (8.7 ± 2.5%), while remaining unchanged in the CTL group (Pre: 8.5 ± 3.5%; Post 2: 9.2 ± 3.4%; *p* = 0.607, *W* = 0.036, Figure 3a). Allometrically scaled FMD exhibited time‐related changes between Pre and Post 2 (*p* = 0.022; Figure 3b) in the IHC group (main effect of time: *p* = 0.023; 𝜂_𝑝_^2^ = 0.150); no alteration was observed in the CTL group. Additionally, no significant changes over time or between‐group differences were observed for time to peak dilation and SR_AUC_ (all *p* > 0.05).

**TABLE 2:** Characteristics of brachial artery flow‐mediated dilation across groups and time points.

	CTL (*N* = 14)			IHC (*N* = 12)		
	Pre	Post 1	Post 2	Pre	Post 1	Post 2
*D*_BASE_ (cm)	0.37 ± 0.063*	0.356 ± 0.057	0.372 ± 0.069*	0.381 ± 0.061	0.378 ± 0.061	0.369 ± 0.06
*D*_PEAK_ (cm)	0.401 ± 0.072*	0.385 ± 0.064	0.407 ± 0.077*	0.403 ± 0.06	0.405 ± 0.063	0.401 ± 0.061
*D*_DIFF_ (cm)	0.032 ± 0.016	0.029 ± 0.013	0.034 ± 0.015	0.022 ± 0.009	0.027 ± 0.013	0.031 ± 0.008
Time to peak (s)	54.4 ± 22.9	58.9 ± 24.4	67.4 ± 37.2	52.2 ± 18.9	61.6 ± 38	61.5 ± 15.3
SR_AUC_ (A.U)	27,042 ± 16,052	29,707 ± 11,105	28,465 ± 11,593	25,303 ± 16,604	24,499 ± 12,359	29,420 ± 16,628

*Note*: Data are mean ± SD. A Friedman's ANOVA and multiple pairwise comparisons (Durbin‐Conover) were used for intra‐group analysis. Between group comparisons were performed using Mann–Whitney tests.

Abbreviations: CTL, control group; *D*_BASE_, baseline artery diameter; *D*_DIFF_, absolute difference in artery diameter; *D*_PEAK_, peak artery diameter; IHC, intermittent hypoxic conditioning group; SR_AUC_, shear rate area under the curve.

* *p* < 0.05 vs. Post 1 for the group CTL.

### 3.3 Nitrate‐mediated dilation

Nitrate‐mediated dilation data are depicted in Table 3. *D*_BASE_ and *D*_PEAK_ remained consistent and similar across all time points in both groups (*p* > 0.05), with no significant differences observed in absolute (*D*_DIFF_) and relative (NMD%) changes in artery diameter, time to peak, and SR_AUC_.

**TABLE 3:** Characteristics of brachial artery nitrate‐mediated dilation across groups and time points.

	CTL (*N* = 14)			IHC (*N* = 11)		
	Pre	Post 1	Post 2	Pre	Post 1	Post 2
*D*_BASE_ (cm)	0.365 ± 0.06	0.364 ± 0.065	0.378 ± 0.081	0.381 ± 0.059	0.386 ± 0.064	0.379 ± 0.054
*D*_PEAK_ (cm)	0.439 ± 0.062	0.436 ± 0.075	0.449 ± 0.088	0.461 ± 0.066	0.463 ± 0.078	0.453 ± 0.064
*D*_DIFF_ (cm)	0.074 ± 0.012	0.072 ± 0.026	0.071 ± 0.017	0.08 ± 0.024	0.077 ± 0.025	0.075 ± 0.019
NMD (%)	20.6 ± 4.7	20.2 ± 7.4	19.3 ± 5.3	21.6 ± 8.3	19.9 ± 6.1	19.8 ± 4.6
Time to peak (s)	378 ± 101	319 ± 124	326 ± 74	348 ± 127	363 ± 89	314 ± 88
SR_AUC_ (A.U)	22,858 ± 9593	34,132 ± 30,339	31,791 ± 20,948	42,745 ± 41,480	32,597 ± 22,943	37,457 ± 30,754

*Note*: Data are mean ± SD. A two‐way repeated‐measures ANOVA was conducted to assess differences between groups and across time points. Data were not available in one subject from the IHC group due to technical reasons.

Abbreviations: CTL, control group; *D*_BASE_, baseline artery diameter; *D*_DIFF_, absolute difference in artery diameter; *D*_PEAK_, peak artery diameter; IHC, intermittent hypoxic conditioning group; SR_AUC_, shear rate area under the curve.

### 3.4 Cardiopulmonary exercise test

Cardiopulmonary exercise testing data are depicted in Table 4. We observed a main effect of time for peak work rate (*p* = 0.049; 𝜂_𝑝_^2^ = 0.118); however, post hoc analysis did not reveal any significant difference across time and groups. Minute ventilation at peak exercise ($\dot{V_{E}}$) increased significantly in the IHC group from Pre to Post 1 (*p* = 0.021; main interaction effect: *p* = 0.005; 𝜂_𝑝_^2^ = 0.197). This was driven by non‐significant increases in both tidal volume (*V*_T_) and respiratory rate (RR) in the IHC group (*V*_T_: *p* = 0.081, 𝜂_𝑝_^2^ = 0.099; RR: *p* = 0.082, 𝜂_𝑝_^2^ = 0.099). Other metabolic, cardiovascular, pulmonary gas exchange and perceptual parameters remained stable across throughout the study.

**TABLE 4:** Physiological and perceptual responses at peak incremental cardiopulmonary exercise testing across groups and time points.

	CTL (*N* = 14)			IHC (*N* = 12)		
	Pre	Post 1	Post 2	Pre	Post 1	Post 2
Metabolic/cardiovascular						
Work rate (W)	131 ± 43	132 ± 48	134 ± 45	138 ± 33	141 ± 33	144 ± 35
Work rate (% pred)	121 ± 26	122 ± 29	124 ± 26	119 ± 19	122 ± 20	126 ± 22
$\dot{V}O_{2\text{peak}}$ (L.min^−1^)	1.88 ± 0.65	1.89 ± 0.66	1.89 ± 0.61	1.84 ± 0.42	1.99 ± 0.50	1.93 ± 0.47
$\dot{V}O_{2\text{peak}}$ (% pred)	134 ± 23	135 ± 24	137 ± 23	126 ± 23	138 ± 18	132 ± 20
$\dot{V}{\mathrm{CO}}_{2}$ (L.min^−1^)	1.97 ± 0.60	1.98 ± 0.64	2.0 ± 0.59	1.94 ± 0.46	2.12 ± 0.46	2.08 ± 0.44
HR (bpm)	155 ± 17	150 ± 16	152 ± 17	161 ± 9	153 ± 13	162 ± 13
HR (% pred)	119 ± 14	116 ± 11	117 ± 13	125 ± 10	118 ± 14	126 ± 14
Ventilatory						
$\dot{V_{E}}$ (L.min^−1^)	82.6 ± 26.1	79.9 ± 24.9	84.1 ± 25.8	72.7 ± 18.9	82.5 ± 19.2*	81.3 ± 18.5
*V*_T_ (L)	2.0 ± 0.67	1.98 ± 0.64	2.01 ± 0.56	1.97 ± 0.44	2.14 ± 0.53	2.03 ± 0.49
RR (Breaths.min^−1^)	41 ± 7	40 ± 5	41 ± 4	37 ± 6	39 ± 7	40 ± 8
Pulmonary gas exchange						
SpO_2_ (%)	96 ± 3	96 ± 1	95 ± 2	95 ± 3	96 ± 2	96 ± 2
PetCO_2_ (mmHg)	31 ± 2	31 ± 2	31 ± 2	34 ± 4	33 ± 5	33 ± 5
Symptoms						
Dyspnea	7 ± 3	8 ± 2	8 ± 2	7 ± 2	8 ± 2	8 ± 2
Leg discomfort	8 ± 2	8 ± 2	9 ± 2	7 ± 2	8 ± 2	8 ± 2

*Note*: Data are presented as mean ± SD. For parametric data, a two‐way repeated‐measures ANOVA was conducted to assess differences between groups and across time points. A Bonferroni correction was applied for the post hoc tests. For non‐parametric data, a Friedman's ANOVA and multiple pairwise comparisons (Durbin‐Conover) were used for intra‐group analysis. Between‐group comparisons were performed using Mann–Whitney tests.

Abbreviations: $\dot{V}{\mathrm{CO}}_{2}$, carbon dioxide output; $\dot{V}O_{2}$, oxygen uptake; $\dot{V_{E}}$, minute ventilation; CTL, control group; EtCO_2_, end tidal carbon dioxide output; HR, heart rate; IHC, intermittent hypoxic conditioning group; RR, respiratory rate; SpO_2_, oxygen saturation by pulse oximetry; VT, tidal volume.

* *p* < 0.05 vs. Pre for the IHC group.

### 3.5 24‐hour ambulatory blood pressure monitoring

Arterial blood pressure data are presented in Table 5. In the IHC group, SBP exhibited a trend toward a decrease during daytime (main effect of time: *p* = 0.070, *η*_*p*_^2^ = 0.105). During nighttime, a significant interaction effect was observed for DBP (main interaction effect: *p* = 0.021, 𝜂_𝑝_^2^ = 0.149) and for MAP (*p* = 0.047, 𝜂_𝑝_^2^ = 0.119). However, post hoc analyses did not reveal statistically significant differences between time points or between groups.

**TABLE 5:** Arterial blood pressure changes across groups and time points.

	CTL (*N* = 14)			IHC (*N* = 12)		
	Pre	Post 1	Post 2	Pre	Post 1	Post 2
24‐h						
SBP (mmHg)	122 ± 15	125 ± 15	122 ± 11	123 ± 12	120 ± 12	118 ± 13
DBP (mmHg)	78 ± 8	78 ± 10	79 ± 8	76 ± 6	74 ± 6	73 ± 7
MAP (mmHg)	93 ± 10	94 ± 12	93 ± 9	91 ± 8	89 ± 8	88 ± 9
Daytime						
SBP (mmHg)	125 ± 15	129 ± 16	126 ± 11	128 ± 14	123 ± 13	122 ± 13
DBP (mmHg)	81 ± 8	82 ± 11	81 ± 9	79 ± 7	76 ± 7	76 ± 8
MAP (mmHg)	96 ± 10	98 ± 13	96 ± 9	95 ± 9	92 ± 9	91 ± 9
Night time						
SBP (mmHg)	109 ± 17	108 ± 13	113 ± 16	108 ± 7	109 ± 11	107 ± 15
DBP (mmHg)	68 ± 12	66 ± 9	71 ± 11	65 ± 6	66 ± 7	63 ± 7
MAP (mmHg)	82 ± 13	80 ± 10	85 ± 12	80 ± 6	80 ± 8	78 ± 9

*Note*: Data are presented as mean ± SD. A two‐way repeated‐measures ANOVA was conducted to assess differences between groups and across time points. A Bonferroni correction was applied for the post hoc tests.

Abbreviations: CTL, control group; DBP, diastolic blood pressure; IHC, intermittent hypoxic conditioning group; MAP, mean arterial pressure; SBP, systolic blood pressure.

## 4 DISCUSSION

To date, studies have only investigated the acute effects of IH on peripheral vascular function in young adults (Hanson et al., 2022; Lewis et al., 2017) and/or healthy elderly individuals (Stray‐Gundersen et al., 2024), frequently highlighting the need to extend research to repeated IH exposure. Consequently, and to the best of our knowledge, this study is the first to examine the effect of passive IHC on vascular function in elderly participants. Our main findings suggest an improvement in peripheral vascular function. However, this was not accompanied by significant changes in other cardiovascular health parameters typically linked to FMD (i.e., CRF and ABP control).

### 4.1 Effects of IHC on peripheral vascular function

Applying allometric scaling to our data revealed a significant time effect with a moderate effect size from Pre to Post 2 following IHC exposure. The reasoning behind this scaling lies in the fact that FMD is calculated as a percentage and is, therefore, directly dependent on the baseline artery diameter. Consequently, to account for *D*_BASE_ variability across groups and time points, we performed allometric scaling in accordance with the methodology described by Atkinson et al. (Atkinson et al., 2013) (see Section 2.6 for further details on the procedure). In the present study, applying this correction is paramount since we did observe a difference in *D*_BASE_ across time in the CTL group.

The potential mechanisms underlying this improvement may be categorized into two key pathways. First, both continuous and intermittent hypoxia exposure have been associated with transient increases in brachial artery blood flow and thus shear stress (Hanson et al., 2022). In this context, it is well known that improvements in vascular function are closely related to mean elevation or fluctuation in antegrade shear rate during intervention (Holder et al., 2019; Tinken et al., 2009). Based on these findings, it may follow that our IHC protocol has produced multiple, repeated in‐session increases in shear stress, thereby contributing to the observed improvement in FMD.

In addition, under hypoxic conditions, HIFs (hypoxia inducible factors) rapidly accumulate, initiating the transcription of numerous target genes involved in key physiological processes such as NO synthesis, a potent vasodilator agent (Burtscher et al., 2024). Intermittent hypoxia is implicated in various clinical conditions including obstructive sleep apnea and tumor progression (Lévy et al., 2015; Saxena & Jolly, 2019). It may appear counterintuitive that such hypoxic stimuli may also lead to beneficial physiological effects. In fact, the beneficial or detrimental impact of hypoxia crucially depends on the exposure pattern (Navarrete‐Opazo & Mitchell, 2014). Severe (2%–8% inspired O_2_) and highly cyclical (48–2400 cycles/day) IH have been associated with deleterious outcomes such as oxidative stress and systemic inflammation. In contrast, moderate intermittent hypoxia (9%–16% inspired O_2_) combined with a low number of cycles (3–15 cycles/day)—that are similar to the present IHC protocol—has been identified as a potentially safe and effective therapeutic strategy (Verges et al., 2015).

Nitrate‐mediated dilation is commonly assessed alongside FMD to differentiate between endothelial cells and vascular smooth muscle cell dysfunction and provides valuable information about maximal vasodilation capacity (Maruhashi et al., 2018). In the present study, two main findings emerged. First, based on the 15.6% dilation cut‐off proposed by Maruhashi et al. (Maruhashi et al., 2020) as indicative of normal vascular smooth muscle function, our participants did not exhibit any sign of vascular smooth muscle impairment at any time point, which could have influenced their sensitivity to nitric oxide (NO) released by endothelial cells. More importantly, our IHC intervention did not seem to elicit any measurable effect on NMD, suggesting that the observed improvements in FMD are likely attributable to changes in endothelial function (e.g., NO bioavailability) rather than vascular smooth muscle responsiveness.

### 4.2 Effects of IHC on relevant outcomes related to vascular function

Arterial blood pressure lowering is arguably one of the most robust physiological outcomes associated with IHC (Serebrovskaya et al., 2008). In our analysis, we observed a mean decrease of 6 ± 9 mmHg in daytime SBP between Pre and Post 2 measurements. While statistically non‐significant, this reduction may still be clinically relevant, especially bearing in mind that our participants were normotensive—or already treated for hypertension (*n* = 2). Indeed, previous research has evidenced that even a modest reduction of 5 mmHg in SBP was associated with a 10% reduction in the risk of a major cardiovascular event (Rahimi et al., 2021).

The mechanisms underlying potential ABP‐lowering effects of IHC have been extensively discussed. Briefly, these effects may be driven by HIFs‐regulated increases in NO bioavailability (Coulet et al., 2003) and modulation of both the sympathetic and parasympathetic nervous systems (Panza et al., 2022). Supporting this assumption, Muangritdech et al. (2020) evaluated the effects of IHC (both at rest and in combination with exercise) in hypertensive patients on various biological markers and ABP. In both groups, they observed a significant inverse correlation between the reduction in SBP and the increase in NO metabolites and HIF‐1α levels measured 2 days after IHC cessation. Interestingly, these authors—and others (Lyamina et al., 2011)—reported that IHC‐induced reductions in ABP remained present up to 28 days after the intervention. This sustained reduction was further associated with a decrease in malondialdehyde concentrations in the combined intervention group, suggesting a persistent attenuation of oxidative stress (Muangritdech et al., 2020).

Given the close relationship between changes in FMD and ABP with oxidative stress (Donato et al., 2007), our results (consistent with observations by Muangritdech et al. (2020) and Lyamina et al. (2011)) suggest that our IHC intervention might have induced durable effects on both NO bioavailability and oxidative stress reduction, which could at least in part explain the continuous improvement in FMD and SBP from baseline measurements to 2 months following the IHC cessation.

While exercise training is well‐known to improve both FMD and CRF (Green et al., 2017), relatively few studies have investigated the effects of IHC on CRF (Lizamore & Hamlin, 2017), especially with passive exposure to hypoxia. For instance, Muangritdech et al. (2020) reported an improvement in the 6‐min walk distance after both passive and exercise‐combined IHC protocols. In combination with moderate exercise training, Chacaroun, Borowik, Iv‐Ey, et al. (2020) demonstrated that IHC led to increases in $\dot{V}O_{2\text{peak}}$ (+10 ± 11%) and in peak $\dot{V_{E}}$ (~11%) in obese individuals. These present findings are of a similar magnitude to the increases both in $\dot{V}O_{2\text{peak}}$ ($\dot{V}O_{2}$: +9 ± 18%; +0.15 ± 0.36 L.min^−1^; N.S.) and peak $\dot{V_{E}}$ ($\dot{V_{E}}$: +15 ± 20%; +9.8 ± 12.2 L.min^−1^; *p* < 0.05) observed herein. In this study, we observed an increase in peak $\dot{V_{E}}$ in the IHC group following the conditioning period. It is important to first acknowledge that an increase—even slight—in maximal exercise intensity, as observed in both groups, could contribute to an elevation in maximal minute ventilation. However, this factor alone is unlikely to fully explain the observed effect, as no similar increase in $\dot{V_{E}}$ was detected in the CTL group. Morevover, hypoxia is well‐known to induce ventilatory adjustments collectively referred as the hypoxic ventilatory response. This response is closely linked to chemosensitivity and can be characterized by sustained increase in $\dot{V_{E}}$, which persist even in the short term after the hypoxic stimulus cessation (Teppema & Dahan, 2010). This mechanism is thought to be mediated by enhanced synaptic pathways between the carotid bodies and respiratory motoneurons (Pamenter & Powell, 2021). Nevertheless, previous studies investigating the effects of chronic IH exposure have yielded mixed results. For instance, Townsend et al. (2005) reported significant increases in ventilation during exercise conducted under normoxic conditions, whereas Foster et al. (2006) observed no such effect. Given that ventilatory adjustments to exercise are at least partially regulated by peripheral chemoreceptors, hypoxia‐induced increases in chemosensitivity may optimize ventilatory responses during exercise. Hence, since chemosensitivity is impaired with aging (García‐Río et al., 2007), the improvements observed after our IHC program might reflect enhanced peripheral chemoreceptor sensitivity.

### 4.3 Limitations and methodological considerations

Owing to the time‐consuming nature of the protocol (3 sessions per week over an 8‐week period), we were able to enroll 31 participants over a ~3‐year period. Our study might thus lack statistical power, and including a larger cohort would have been beneficial, particularly as several outcomes exhibited trends toward significance. Moreover, given known sex‐related differences in both vascular function (Seeland et al., 2021) and responses to hypoxia (Raberin et al., 2024), including a dedicated sample of women to allow for sex‐stratified analyses would be of considerable interest. Overall, future studies with a larger sample size are thus needed to confirm and expand upon these results.

In line with other studies, we suspected an IHC‐induced, HIFs‐mediated increase in NO bioavailability. However, the absence of biological sampling precludes direct confirmation of such a mechanism. Incorporating these measurements in future studies would allow for an objective assessment of this pathway. Additionally, we also assumed that repeated, in‐session increases in peripheral blood flow and shear stress contributed to the observed enhancements in vascular function, but these measurements were not performed in the present study.

Finally, longitudinal assessments of FMD require careful methodological considerations, and we followed as closely as possible the recommendations described by Thijssen et al. (2019). We, however, acknowledged the practical challenges imposed by participants' availability, and every effort was made to minimize deviations from these recommendations (e.g., investigations conducted at similar times of the day for a given participant).

### 4.4 Conclusion and significance

This study is the first to investigate the effects of passive, repeated IHC on peripheral endothelial function in elderly individuals. We observed that the latter numerically increased over the 8‐week period and was even significantly improved 2 months after IHC cessation. Additionally, we reported significant and near‐significant improvements in cardiorespiratory and blood pressure parameters, the latter potentially being of clinical relevance. These results suggest that a well‐calibrated IHC protocol may contribute to reducing cardiovascular risk in elderly individuals. Overall, this study reinforces evidence supporting IHC as a low‐risk intervention and a valuable therapeutic strategy for improving relevant health‐related parameters.

Future studies may investigate the combined effects of IHC and exercise training on these outcomes.

## AUTHOR CONTRIBUTIONS

S.D., J.V.B., S.V., and M.M. conceived and designed research; H.R., T.P.P., A.G., D.K., J.V.B., and M.M. performed experiments; H.R. and T.P.P. analyzed data; H.R. prepared figures; H.R. and M.M. drafted manuscript; H.R., T.P.P., A.G., D.K., P.F., B.C., M.G., S.D., J.V.B., S.V., and M.M. edited, revised, and approved final version of manuscript.

## FUNDING INFORMATION

This work was supported by the French National Research Agency in the framework of the “Investissements d'avenir” program (ANR‐15‐IDEX‐02).

## CONFLICT OF INTEREST STATEMENT

The authors declare they have no conflicts of interest, whether financial or otherwise.

## ETHICS STATEMENT

This study received approval from a national ethics committee (CPP Ouest VI, No. ID/RCB: 2020‐A03582‐37) and was registered at clinicaltrials.gov (*NCT05048680*). All participants were fully informed of the procedures and risks and gave their written consent prior to study participation.

## Data Availability

Data will be provided upon reasonable request to the corresponding author.
